# Initial biological classification of Lewy body diseases: No consensus on terminology

**DOI:** 10.1002/alz.14449

**Published:** 2024-12-28

**Authors:** Anthony E. Lang, Günter U. Höglinger, Charles H. Adler, Daniela Berg, Christine Klein, Tiago Fleming Outeiro, Werner Poewe, Ron Postuma, A. Jon Stoessl

**Affiliations:** ^1^ Edmond J. Safra Program in Parkinson's Disease and the Rossy PSP Centre Toronto Western Hospital and the University of Toronto Toronto Ontario Canada; ^2^ Department of Neurology, LMU University Hospital Ludwig‐Maximilians‐Universität (LMU) Munich Germany; ^3^ German Center for Neurodegenerative Diseases (DZNE) Munich Germany; ^4^ Munich Cluster for Systems Neurology (SyNergy) Munich Germany; ^5^ Mayo Clinic Alix School of Medicine Mayo Clinic Arizona Scottsdale Arizona USA; ^6^ Christian‐Albrechts‐University and University Hospital Schleswig‐Holstein, Campus Kiel Kiel Germany; ^7^ Schilling Professor of Neurogenetics and Neurology, Director of the Institute of Neurogenetics University of Luebeck and University Hospital Schleswig‐Holstein Lüebeck Germany; ^8^ University Medical Center Göttingen, Department of Experimental Neurodegeneration Center for Biostructural Imaging of Neurodegeneration Göttingen Germany; ^9^ Translational and Clinical Research Institute, Faculty of Medical Sciences Newcastle University Newcastle Upon Tyne UK; ^10^ Medical University Innsbruck Innsbruck Austria; ^11^ Department of Neurology and Neurosurgery The Neuro (Montreal Neurological Institute‐Hospital), McGill University Montreal Quebec Canada; ^12^ Djavad Mowafaghian Centre for Brain Health Faculty of Medicine University of British Columbia Vancouver British Columbia Canada

1

To the Editor,

We read with considerable interest the revised criteria for the diagnosis and staging of Alzheimer's disease (AD) by Jack et al.^1^ This summarizes the long history and multi‐staged development and evolution of biological concepts underlying their updated proposal. Given the frequency of Lewy body co‐pathology in AD, we agree with the inclusion of alpha synuclein as a biomarker for non‐AD co‐pathology in AD, especially given its potential impact on the clinical course (e.g., Levin et al.^2^). Conversely, we also propose to consider amyloid beta and tau as potential co‐pathology which may be of relevance in biologically defined Parkinson's disease (PD).^3^ However, when discussing biomarkers for alpha synuclein,^1^ the authors state that PD and dementia with Lewy bodies (DLB) “have recently been re‐labeled as NSD” (NSD: “neuronal synuclein disease”), and this is repeated throughout the article. We wish to highlight that the term “NSD” is not currently an accepted designation by the PD or DLB communities. This terminology and concept have not been carefully evaluated in the way AD proposed criteria have. The term NSD and its accompanying integrated staging system have only recently been proposed in a single article,^4^ and have not been sufficiently considered, vetted, and supported by large segments of the research community dealing with alpha synuclein–related diseases (the community at large currently rather uses the term “Lewy body disorder” or “Lewy body disease” as the umbrella term for PD and DLB^5^, ^6^ and we have also argued that the diagnosis of PD should not be restricted to those with Lewy pathology).^3^ Indeed, this proposal has engendered considerable discussion and controversy in the field, and disagreement has already been registered from researchers in the areas of rapid eye movement behavior disorder,^7^ dementia with Lewy bodies^8^ and neuropathology,^9^ as well as the International Parkinson and Movement Disorder Society.^10^ We are at the earliest stages in the development of a biological classification/definition of Lewy body diseases, and, until the field can reach greater consensus, we consider that it would be unwise to portray, accept, or endorse the re‐labeling of PD and DLB as NSD by the AD community. This is particularly relevant given the proposal for including alpha synuclein seed amplification assays as a biomarker for non‐AD co‐pathology.

## CONFLICT OF INTEREST STATEMENT

The authors declare no conflicts of interest. Author disclosures are available in the supporting information.

## FUNDING INFORMATION

Nothing relevant to this letter.

## Supporting information



Supporting Information
